# Electrocommunication signals indicate motivation to compete during dyadic interactions of an electric fish

**DOI:** 10.1242/jeb.242905

**Published:** 2021-10-06

**Authors:** Till Raab, Sercan Bayezit, Saskia Erdle, Jan Benda

**Affiliations:** 1 Institute for Neurobiology, Neuroethology Lab, Eberhard Karls Universität, 72076 Tübingen, Germany; 2 Centre for Integrative Neuroscience, Eberhard Karls Universität, 72078 Tübingen, Germany; 3 Bernstein Centre for Computational Neuroscience, Eberhard Karls Universität, 72078 Tübingen, Germany

**Keywords:** Staged competition, Resource holding potential, Assessment, Communication, Weakly electric fish

## Abstract

Animals across species compete for limited resources. Whereas in some species competition behavior is solely based on the individual's own abilities, other species assess their opponents to facilitate these interactions. Using cues and communication signals, contestants gather information about their opponent, adjust their behavior accordingly, and can thereby avoid high costs of escalating fights. We tracked electrocommunication signals known as ‘rises’ and agonistic behaviors of the gymnotiform electric fish *Apteronotus leptorhynchus* in staged competition experiments. A larger body size relative to the opponent was the sole significant predictor for winners. Sex and the frequency of the continuously emitted electric field only mildly influenced competition outcome. In males, correlations of body size and winning were stronger than in females and, especially when losing against females, communication and agonistic interactions were enhanced, suggesting that males are more motivated to compete. Fish that lost competitions emitted the majority of rises, but their quantity depended on the competitors’ relative size and sex. The emission of a rise could be costly since it provoked ritualized biting or chase behaviors by the other fish. Despite winners being accurately predictable based on the number of rises after the initial 25 min, losers continued to emit rises. The number of rises emitted by losers and the duration of chase behaviors depended in similar ways on physical attributes of contestants. Detailed evaluation of these correlations suggests that *A. leptorhynchus* adjusts its competition behavior according to mutual assessment, where rises could signal a loser's motivation to continue assessment through ritualized fighting.

## Abstract (precis)

**Summary:** Electric fish adjust their competition behavior according to mutual assessment, where electrocommunication with so-called ‘rises’ could signal a loser's motivation to continue assessment through ritualized fighting.

## INTRODUCTION

Across animal species, fighting is a key behavior to secure access to limited resources (Clutton-Brock et al., 1979; Chapman et al., 1995; Markham et al., 2015). However, competition is costly because of the energy and time allocated to it, and the increased risk of injury or death (e.g. Briffa and Elwood, 2004). Therefore, individual behavioral decisions during contests are strongly dependent on the associated potential benefits and costs (Arnott and Elwood, 2008, 2009). Often, the best predictor for the outcome of competitions is the contestants’ fighting ability, also called resource holding potential (RHP; Parker, 1974). Usually, larger and stronger individuals win contests, because their physical advantages (higher RHP) directly reflect their increased endurance and potential to inflict damage (Archer, 1988). Additional factors such as weaponry, experience and sex, or positional advantages also influence RHP (reviewed in Arnott and Elwood, 2008).

Behaviors and the course of competition have been shown to be either based on the assessment of solely the individual's own RHP or by integrating both the individual and opponent's RHP (Taylor et al., 2001; Enquist et al., 1990; Huyghe et al., 2005). In the first case (self-assessment), costs resulting from competition are accumulated until an endurance threshold, set by an individual's RHP, is reached and the respective individual retreats (Arnott and Elwood, 2009). Competition costs either arise exclusively from an individual's own behaviors (pure self-assessment, Taylor and Elwood, 2003) or are supplemented by costs inflicted by opponents (cumulative assessment; Payne, 1998). In both cases, no direct information about an opponent and its RHP is gathered. Alternatively, in ‘mutual assessment’, the contestants assess each other's RHP, compare it to their own, and adjust their behavior according to the difference (Enquist and Leimar, 1987). The huge benefit of this strategy is its economic efficiency. Individuals can recognize their inferiority and retreat long before their endurance threshold is reached, thereby saving metabolic costs for both competitors.

Besides passive cues signaling RHP, actively produced communication signals may facilitate interactions during animal conflict (Arnott and Elwood, 2009; Seyfarth et al., 2010). They can directly indicate and reflect an individual's RHP (Davies and Halliday, 1978; Clutton-Brock et al., 1979), but also convey additional information influencing contest and its outcome, like motivation and behavioral intent (e.g. aggression: Kareklas et al., 2019; Westby and Box, 1970; or submission: Hupé and Lewis, 2008; Batista et al., 2012) or social status (Fernald, 2014). Such low-cost signals have been shown to reduce the intensity and duration of contests or even convey sufficient information to resolve conflicts without the necessity of physical competitions (Parker, 1974; Clutton-Brock et al., 1979; Janson, 1990).

To prevent high costs of repetitive fights with the same opponents, dominance hierarchies are established in various species (Creel et al., 1996; Janson, 1985; Clutton-Brock et al., 1979). In dominance hierarchies the necessity of fighting is reduced since access to resources is regulated through social status, favoring those individuals of higher rank (Wauters and Dhondt, 1992; Taves et al., 2009). The organization and characteristics of dominance hierarchies vary across species (Janson, 1985; Cigliano, 1993; Sapolsky, 2005). While in group-living species complex social structures, such as a leader–follower dynamic, can emerge (Strandburg-Peshkin et al., 2018), in solitary species, dominance is rather associated with resource-based benefits, such as the occupation of higher quality territories and increased reproductive success (e.g. Cigliano, 1993). Differences in the abundance and dispersion of food can further lead to variations regarding the skewness in access to resources across social ranks. In bottom-up egalitarian hierarchies, resources are more equally distributed (Sapolsky, 2005), whereas in top-down despotic hierarchies, access to resources is strongly skewed in favor for dominant individuals (Kappeler and Schäffler, 2008).

Dominance hierarchies have also been suggested for the nocturnal gymnotiform electric fish *Apteronotus leptorhynchus* (Dunlap and Oliveri, 2002; Stamper et al., 2010; Raab et al., 2019). *A. leptorhynchus* competes for mates only during the mating season (Hagedorn and Heiligenberg, 1985; Henninger et al., 2018), at other times they compete for optimal shelters (Dunlap and Oliveri, 2002). The corresponding competitions are characterized by ritualized fighting behaviors accompanied by electrocommunication signals (Triefenbach and Zakon, 2008; Smith, 2013). While body size has been shown to be the main determinant for the outcome of competitions in gymnotiformes (Batista et al., 2012; Triefenbach and Zakon, 2008; Dunlap and Oliveri, 2002), the influence of other factors such as sex and communication signals, require further investigation.

Electric signaling has been shown to be an integral aspect of agonistic behaviors in gymnotiform fish (Westby and Box, 1970; Batista et al., 2012; Hupé and Lewis, 2008; Henninger et al., 2018). The frequency of their continuous electric organ discharge (EOD) has been suggested to signal an individual's physical condition, dominance status or aggressiveness (Westby and Box, 1970; Hagedorn and Heiligenberg, 1985; Cuddy et al., 2012). The sexually dimorphic EOD frequency (EODf) of *A. leptorhynchus* indicates identity and sex (Henninger et al., 2020), with males having higher EODfs than females (Meyer et al., 1987). While some studies also suggest that higher EODf indicates dominance (Hagedorn and Heiligenberg, 1985; Dunlap and Oliveri, 2002; Henninger et al., 2018; Raab et al., 2019), others were not able to replicate this correlation (Triefenbach and Zakon, 2008).

For generating distinct electrocommunication signals, electric fish modulate their EODf on various time scales (Benda, 2020). So-called ‘chirps’ are several types of brief (10–500 ms), transient increases in EODf (Engler et al., 2000; Zakon et al., 2002; Hupé et al., 2008). ‘Small’ and ‘long’ chirps are used in courtship for synchronizing spawning (Henninger et al., 2018) and at the same time the very same small chirps are used as submissive signals to reduce attacks in agonistic encounters (Hupé and Lewis, 2008; Henninger et al., 2018). Another category of electrocommunication signals, so-called ‘rises’, are characterized by a moderate increase in EODf by no more than a few tens of Hertz followed by an approximately exponential decay back to baseline EODf from within a second up to almost a minute (Hupé and Lewis, 2008; Henninger et al., 2018). The function of rises is still controversial. They have been suggested to signal aggression or motivation to attack (Tallarovic and Zakon, 2005; Triefenbach and Zakon, 2008), submission (Hopkins, 1974; Serrano-Fernández, 2003), ‘victory cries’ (Dye, 1987), to evoke or precede attacks (Hopkins, 1974; Triefenbach and Zakon, 2008), or to simply be a general expression of stress (Smith, 2013). An enhancement of sensory acquisition by rises is highly unlikely, because the small increase in EODf only marginally influences encoding in electroreceptor afferents (Walz et al., 2014).

Using recently developed techniques for tracking electrocommunication signals in freely behaving electric fish (Henninger et al., 2018, 2020; Madhav et al., 2018) in addition to infrared video recordings, we recorded electric and physical interactions of pairs of *A. leptorhynchus* in staged competitions over a superior shelter. Compared with previous studies we significantly expanded the observation times (from 10 min to 6 h) and the number of interacting pairs of fish. We evaluated the influence of body size, weight, sex and EODf on the outcome of competitions. By analyzing the relationships between rises, agonistic interactions and physical difference between competitors we were able to uncover the fish's assessment strategy, quantify behavioral difference between the sexes and identify the potential uses of rises of *A. leptorhynchus* during competitions.

## MATERIALS AND METHODS

### Animals

A total of 21 mature *Apteronotus leptorhynchus* (Ellis 1912) (9 males, 12 females) not in breeding condition, obtained from a tropical fish supplier, were used. Fish were selected randomly from multiple populations to reduce familiarity effects and sorted into four mixed-sex groups of five or six fish (males/females: group 1: 2/4; group 2: 1/4; group 3: 3/2; group 4: 3/2). Males were identified by their higher EODf (see below) and elongated snout. The sex of one-third of the fish was verified after the competition experiments via post-mortem gonadal inspection in the context of electrophysiological experiments (approved by Regierungspräsidium Tübingen, permit no. ZP 1/16) which verified sex assignments for most of the fish with EOD frequencies close to the male–female cut-off of 740 Hz (Fig. S1A). Fish were housed individually in 54 liter tanks with a 12 h:12 h light:dark cycle prior to the experiments and in between competition trials. Each tank was equipped with a plastic tube for shelter, a water filter and an electrical heater. Water temperature was constant at 25±0.5°C and water conductivity was 200 μS cm^−1^. Fish were fed frozen *Chironomus plumosus* daily. The competition experiments complied with national and European law and were approved by the Regierungspräsidium Tübingen (permit no. ZP 04/20 G).

### Set-up

The competition experiments were run in a 100 liter tank equipped with a 10 cm long and 4 cm wide PVC half-tube as a superior shelter in the center, surrounded by four additional, less optimal shelters (two 5 cm long, 4 cm diameter PVC half-tubes and two 3×5 cm tables, Fig. S1C). Water temperature and conductivity as well as light:dark cycle were identical to those in the housing tanks. A heating mat was placed below the tank and powered with DC current. Two air-powered water filters were placed behind PVC boards with netted windows in the corners of the tank to avoid offering additional shelter. 15 monopolar electrodes at low-noise buffer headstages were mounted on the bottom of the tank. The reference electrode was placed behind a PVC board in one corner of the tank. Electric signals were amplified (100× gain, 100 Hz low-pass filter, EXT-16B, Npi electronic, Tamm, Germany) digitized at 20 kHz per channel with 16 bit resolution (USB-1608GX-2AO, Measurement Computing) and stored on 64 GB USB sticks using custom written software running on a Raspberry Pi 3B. Water temperature was measured every 5 min (Dallas DS18B20 1-wire temperature sensor). Infrared videos were recorded at 25 frames s^–1^ with a camera (Allied Vision Guppy PRO) mounted on top of the tank for all trials of groups 3 and 4. The tank was continuously illuminated by 2×4 infrared lights (ABUS 840 nm) located on the long sides outside the tank. For the synchronization of video and electric recordings we used LED-light pulses of 100 ms duration triggered by the computer-amplifier system in intervals of 5 s. The LED was mounted on the edge of the tank not perceivable by the competing fish, but detectable in the video recordings. The tank, camera and lights were placed inside a Faraday cage.

### Experimental procedure

In each competition trial two fish were freely swimming and interacting in the experimental tank for 6 h. Participating fish were taken from their housing tanks and simultaneously released into the experimental tank. The first 3 h of each trial took place during the dark phase and the second 3 h during the light phase of the circadian rhythm the fish were accustomed to. This limited the experiment to one trial per day. The winner of each trial was identified by its presence within the superior shelter during the light-phase of the trial. Fish were transferred back into their housing tanks after the trial. Pairings for each trial were selected systematically to (i) ensure all possible combinations within each group to be tested (10 combinations for groups of five fish, 15 for the group with six fish), (ii) keep the experience level for all fish equal, and (iii) prevent a single fish from being tested on two consecutive days. Weight and length (body size) of each fish was assessed once a week starting in the week before the competition trials.

With 21 fish in four groups we ended up with a total of 45 pairings and trials. Technical failure led to loss of the electric recordings for the initial four trials of group 4. In another three trials, we were unable to extract EODf traces and electrocommunication signals from the electric recordings because the EODf difference between fish were too low (<0.5 Hz). In a single trial, which we discuss separately, we were unable to determine the winner, because both fish shared the superior shelter at the end of the trail. The remaining 37 trials were analyzed in detail.

### Preprocessing of electric and video recordings

After computing spectrograms for each channel with fast Fourier transformation (FFT, *n*_fft_=2^15^, corresponds to 1.63 s, 80% overlap) we first detected peaks in a power spectrum summed over the channels and assigned them to the fundamental EODfs and their harmonics of the two fish. Based on EODfs and the distribution of power in the channels, we tracked electric signals over time and obtained EODf traces for each of the two fish (Henninger et al., 2018, 2020; Madhav et al., 2018).

To assess baseline EODf, we computed the 5th percentile of non-overlapping, 5 min long EODf trace snippets. EODf is sensitive to temperature changes, which were inevitable throughout the single trials and averaged at 1°C. We computed the *Q*_10_ values resulting from temperature and EODf differences of each 5 min snippet and used the median of 1.37 over all fish to adjust each fish's EODf to 25°C (EODf_25_). The EODf_25_ was used to assess an individual's sex, with EODf_25_>740 Hz assumed to originate from males. As noted above, the sex of half of the fish was verified via post-mortem gonadal inspection.

EODf difference for each pair of fish was estimated from the difference of the median baseline EODfs of the competitors during the light-phase of each trial, where EODfs stayed comparably stable. Rises were identified by detecting their characteristic onset peak in each EODf trace, based on a minimum difference of 5 Hz between a peak and the preceding trough (Todd and Andrews, 1999). Then, the size of the rise, its maximum increase in EODf, was calculated by subtracting the baseline EODf from this peak frequency.

We manually extracted two categories of agonistic interactions from the infrared video recordings using the event logging software BORIS (Friard and Gamba, 2016). For agonistic interactions without physical contact that were characterized as high velocity, directed movements towards a competitor (e.g. chase behavior), we recorded onset and end times. Agonstic physical contacts between competitors such as ritualized biting or head bumping were detected as point events.

### Data analysis

The recorded data and custom analysis scripts are available on request. Data were analyzed in Python version 3.6.8 using numpy (van der Walt et al., 2011), scipy (Oliphant, 2007) and matplotlib (Hunter, 2007) packages. All averages are given with their standard deviation. Mann–Whitney *U*-tests were used to assess significance of differences between two populations and Pearson's test and correlation coefficient *r* for assessing correlations. The influence of various factors on competition outcome was quantified by paired *t*-tests and by the area under the curve (AUC) of a receiver-operating characteristics (ROC). Generalized linear models (GLM):(1)
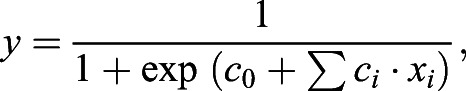


with a logistic link function were used to estimate the combined effects of several factors *x*_*i*_ (continuous: EODf, size, ΔEODf and Δsize, categorical: sex), linearly combined with coefficients *c_i_* and an offset *c*_0_, on the outcome of the competitions *y* (winner or loser). The performance of the GLMs was again assessed by the AUC of a ROC analysis. Standard deviations of AUC values were obtained by 1000 times bootstrapping the data.

To evaluate the influence of the contestants’ physical attributes on the quantity of emitted rises throughout a trial, simple correlations were supplemented by multiple linear regression models. For each model we performed backwards elimination model selection with an elimination criterion of α>0.05.

Temporal coupling between rises and agonistic interactions was quantified by a cross-correlation analysis, i.e. by estimating the averaged rate of rises centered on agonistic events (onset of chase behaviors or physical contact). For this the temporal differences, Δ*t*, between agonistic onsets and rises up to ±60 s were convolved with a Gaussian kernel with a standard deviation of 1 s. Statistical significance was assessed by a permutation test. The null hypothesis of rises not being correlated with agonistic interaction events was obtained by computing the cross-correlation as described above from 1000 random variants of shuffled rise intervals. From this distribution we determined the 1st and 99th percentiles. In addition, we computed the 98% confidence interval for the estimated cross-correlation by 1000 times jack-knife resampling where we randomly excluded 10% of the rises.

We used a time window 5 s prior to agonistic onsets to quantify the average number of rises per agonistic event and to compare them with the corresponding time fractions, the number of agonistic events multiplied with the 5 s window relative to the total dark period time of 3 h. The time window of 5 s was chosen to approximately cover the time of significantly elevated rise rates that we observed before the agonistic events in the cross-correlation analysis.

## RESULTS

In 37 trials we observed and analyzed pairs of *A. leptorhynchus* (6 male pairs, 10 female pairs and 21 mixed-sex pairs) competing for a superior shelter. The 9 males differed from the 12 females by their higher EOD frequency as expected from the sexual dimorphism in EODf in *A. leptorhynchus*. Fish size ranged from 9 to 19 cm and was independent of sex (*U*=51.5, *P*=0.44; Fig. S1A). Fish size strongly correlated with body weight (3.3–20.3 g, *r*=0.94, *P*<0.001; Fig. S1B) and we therefore excluded weight from the following analysis.

**Fig. 1.**
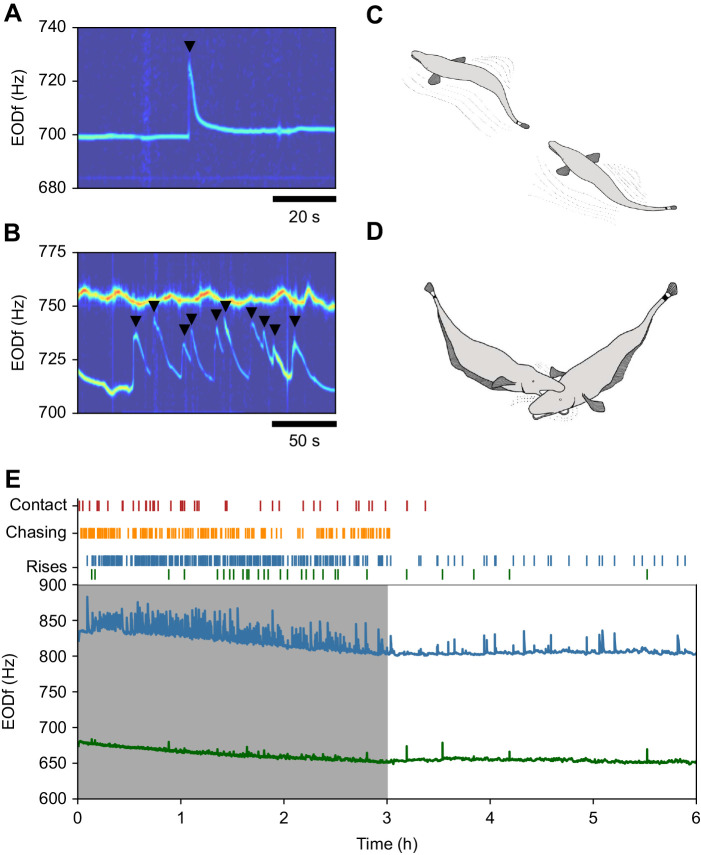
**Behaviors and interactions of *Apteronotus leptorhynchus* during a typical competition trial.** (A) Spectrogram of an electric recording comprising the EODf trace of a fish while emitting a rise as communication signal. Rises are abrupt increases in EODf followed by an exponential decay back to baseline EODf. Rises were detected using their characteristic onset peak (black triangle). (B) Spectrogram of a 200 s section of an electric recording comprising EODf traces of two fish. In the lower EODf trace a series of 10 rises can be seen. (C,D) Ritualized agonistic interactions in *A. leptorhynchus* comprise non-physical chasing (C) and short physical agonistic interactions such as biting or head bumping (D). Both were initiated by fish later winning a trial. (E) *A. leptorhynchus* continuously emits EODs with an individual specific frequency. EODf traces of both competing fish (blue male and green female, bottom panel), time points of physical contacts, onsets of chase behavior, and detected rises (top panels) recorded during the full 6 h trial with the first 3 h in darkness (gray) and the last 3 h during light.

We were able to track electric behaviors of the competitors based on the individual-specific EODf traces, including the detection of rises (electrocommunication signals, Fig. 1A,B). Complementary infrared video recordings obtained during the 20 trials of group 3 and 4 were used to detect ritualized agonistic behaviors, i.e. chasing (Fig. 1C) and physical contacts such as biting or head bumping (Fig. 1D). In a typical competition trial (Fig. 1E), the competing fish's overall activity was much higher during the initial, 3 h dark phase as demonstrated by the higher rates of agonistic interactions and rise emission. During the subsequent 3 h light phase, the activity ceased almost entirely and one fish spent substantially more time within or closer to the superior shelter (99.27±0.006%). This fish was identified as the winner. EODfs of the two fish usually differed clearly and decreased over the course of the experiment because of slightly decreasing water temperature.

### Larger fish win competition

**Fig. 2.**
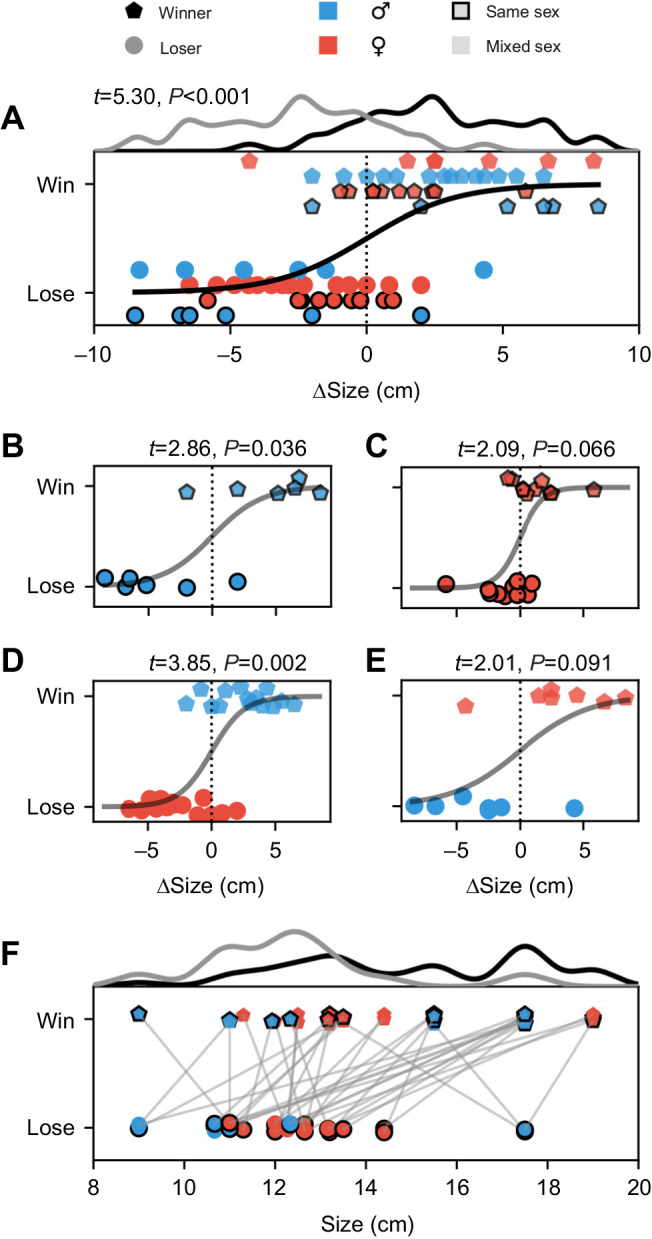
**Body size and size difference of winners and losers.** (A) Winners are larger than their opponents as indicated by a logistic fit and corresponding kernel histograms. (B–E) Winners are larger in all sex pairings and this effect was more distinct for winning males (B,D). (F) Distributions of absolute body size of winners and losers largely overlap. Gray lines connect pairs competing in a trial. Colors and marker style indicate different pairings and the outcome of competitions. Each competition trial contributes two data points, one for the winner and one for the loser. Blue represents males, red females. Pentagons indicate winners, circles losers. Black marker edges indicate same-sex pairings. Winners and losers of each sex pairing are offset in panel A and jittered in panels B–F.

The larger fish of each pairing was more likely to win the competition (*t*=5.3, *P*<0.001; Fig. 2A). Winners are correctly assigned with a probability of 90% based on size difference (in the sense of the AUC of a ROC analysis, Fig. S3C) In particular, in trials won by males, most of the winners were larger than the losers (male–male: 5 out of 6, *t*=2.9, *P*=0.036; male–female: 11 out of 14, *t*=3.9, *P*=0.002; Fig. 2B,D). In trials won by females, this influence of size difference was similarly pronounced but not significant (female–female: 8 out of 10 winners were larger, *t*=2.1, *P*=0.07; female–male: 6 out of 7 winners were larger, *t*=2.0, *P*=0.09; Fig. 2C,E). In 12 of the 21 mixed-sex pairings the males were larger than the competing females, of which only a single male lost. Of the nine larger females three lost. Absolute size, in contrast, did not predict competition outcome (AUC=67%; Fig. 2F). Note that EODf did not correlate with size in either males or females (males: *r*=0.47, *P*=0.20; females: *r*=0.04, *P*=0.90) and that size was independent of sex (Fig. S1A).

### Fish with higher EODf seem to win competitions

Previous studies suggest EODf to be an indicator for dominance in *A. leptorhynchus* (Dunlap and Oliveri, 2002; Henninger et al., 2018; Raab et al., 2019). Indeed, winning fish had on average higher EODfs in comparison to their opponents (*t*=2.1, *P*=0.040; Fig. S2A). However, in same sex pairings analyzed separately, EODf did not predict competition outcome in either males (*t*=0.79, *P*=0.47; Fig. S2B) or in females (*t*=1.4, *P*=0.19; Fig. S2C), but the positive coefficient of the logistic regressions suggests a mild influence of EODf on the outcome of competitions. Furthermore, in mixed-sex pairings, winning males always had higher EODfs and winning females lower EODfs than their opponent, because of the sexual dimorphic EODfs in *A. leptorhynchus* (Fig. S2D,E). These mixed-sex competitions were more often won by males than by females (14 out of 21, Binomial test 14 or more males winning assuming equal chances for both sexes: *P*=0.10). This asymmetry results in an AUC=82% for discriminating winners from losers based solely on sex as a rough proxy for EODf. Although the difference in EODf potentially contains more detailed information than sex alone, it does not discriminate winners from loser better than sex (AUC=75%). Absolute EODf is even less informative about competition outcome (AUC=65%; Fig. S2F).

### Factors influencing competition outcome

We constructed a generalized linear model (GLM, Eqn 1), predicting the competition outcome based on all measured physical factors (size, size difference, EODf, EODf difference and sex; Fig. S3A–C). As expected from the single-factor analysis, size difference is the only factor significantly contributing to the prediction of winners (*t*=2.4, *P*=0.017; Table 1). The model correctly predicts the outcome of 34 of the 37 competition trials with an AUC of 94% (Fig. S3C). Two-factor GLMs based on size differences and either sex or EODf differences perform similarly well (AUC=93%) and slightly better than size difference alone (AUC=90%), further questioning the role of EODfs in predicting competition outcome (Fig. S3C). The outcomes of competitions were independent of previous encounters. Auto correlations of win–lose histories did not differ from those of random sequences, where winners and losers were assigned randomly (permutation test).

**Table 1.**
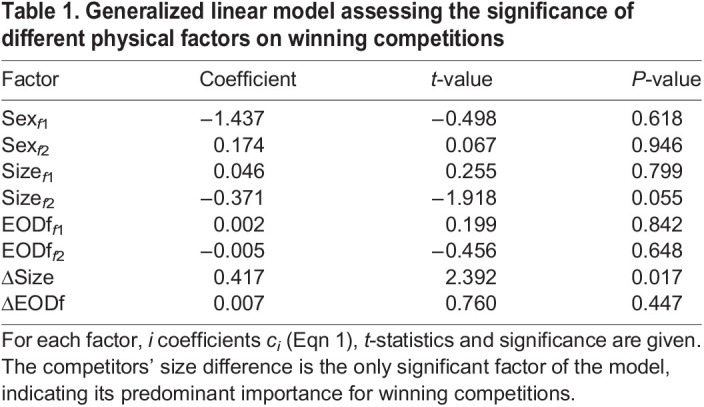
Generalized linear model assessing the significance of different physical factors on winning competitions

### Rises

We detected in total 8530 rises using their characteristic onset EODf peak (Fig. 1A,B). The ‘size’ of rises, the peak EODf relative to baseline EODf before, ranged from the detection threshold of 5 Hz up to 68 Hz with a mean of 17 Hz. We were not able to detect any dependency of our results on the size of rises. In the following we therefore focus on an analysis of their quantity and timing.

### Losing fish emit more rises during the active phase

**Fig. 3.**
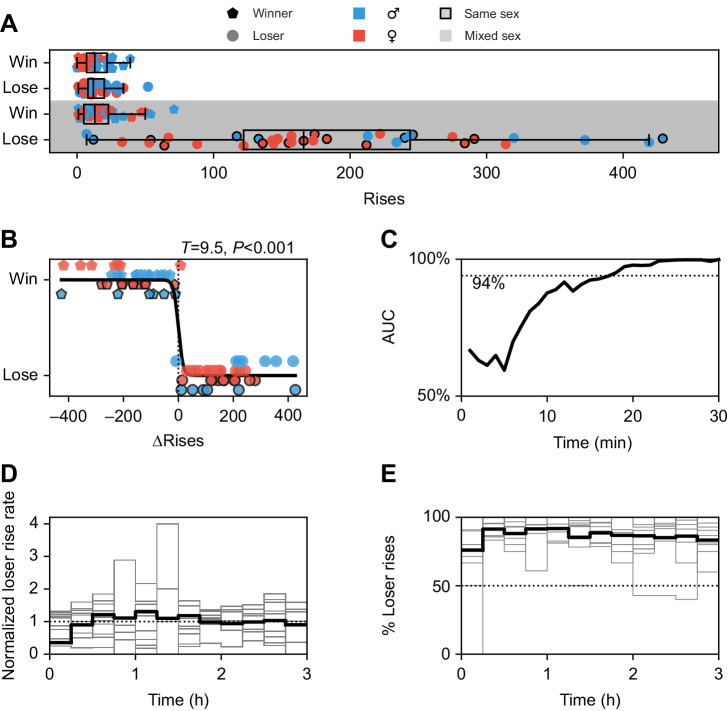
**Rise rates of winners and losers.** (A) Rises are predominantly produced by losers of competitions during the dark phase (shaded). Winners during the dark phase produced equally few rises as both winners and losers during the light phase (white background). (B) Losers of competitions reliably produced more rises than winners in the dark, although absolute and relative numbers of rises varied considerably between trials. (C) Time-resolved discrimination performance between winners and losers based on differences in cumulative rise counts for the first 30 min of the trials. The area under the curve (AUC) of receiver-operating characteristics (ROC) analysis asymptotes to almost 100% after ∼25 min, indicating the outcome of the trials to be already determined the latest from this time on. For comparison, the dashed line at 94% indicates the performance of the GLM including all physical characteristics of the fish from Fig. S3C. (D) Time course of loser rise rates during the dark phase. Rise rates of each trial (gray) were normalized to their mean rate. On average (black), rise rates reached a constant level after ∼30 min. (E) Fraction of rises of losing fish quantified in 15 min time windows is consistently larger than 50% (dashed line) throughout the whole dark period. Individual trials in gray, average over trials in black. Boxes indicate 25th and 75th percentiles with median; whiskers extend to the most extreme value within 1.5×IQR outside the box.

Rises were primarily emitted during the dark phases, i.e. when fish were active (*t*=6.7, *P*<0.001). Fish that later in the light-phase did not occupy the superior shelter, produced 10-fold more rises in the dark phase (184±105) than their winning opponents (18±17, *t*=9.5, *P*<0.001; Fig. 3A). Loser rise counts were highly variable. They ranged from 0 to 419 rises per trial with a coefficient of variation (CV) of 0.63.

The difference between winners and losers in quantitative rise emission during the dark phase almost perfectly predicts winners (AUC=99.9%; Fig. 3B). Initially, discrimination performance exponentially increases from chance level to maximum discrimination starting 5 min after the beginning of a trial with a time constant of ∼5 min (Fig. 3C). The prediction level of 94% based on the physical factors (Fig. S3) is clearly surpassed after ∼20 min. In that time losing fish emitted on average 7.0±5.7 rises and winners just 1.2±1.2.

The losing fish kept emitting higher numbers of rises than the winning fish throughout the dark phase of trials (Fig. 3D). In none of the trials did the competitors switch this behavior (Fig. 3E). The few instances where the fraction of rise counts of losing fish fell below 50% were time windows containing very few rises.

Because of the low numbers of rises produced during the day and by winning fish, we focus in the following on rises produced by losing fish during the night. As detailed below, the number of rises produced by losing fish were dependent on the competitor's sex, their physical differences and the number of trials the fish had already participated in. In contrast, we found no such dependencies for the number of rises emitted by winning fish.

### Losers against females emit more rises

**Fig. 4.**
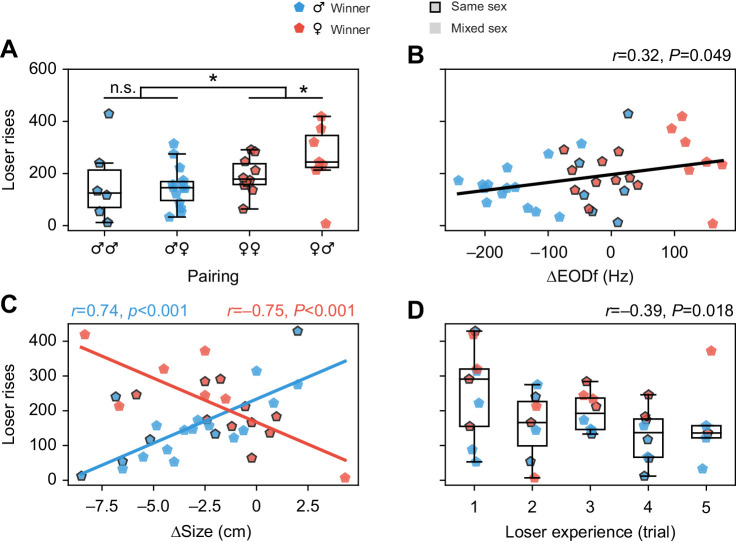
**Dependence of rise counts of losing fish on physical differences between competitors and experience.** (A) In trials won by females (red), losers emit slightly more rises than in trials won by males (blue). The first symbol in pairing categories indicates the winner's sex. (B) With increasing EODf difference to the winning fish (ΔEODf=EODf_loser_−EODf_winner_), losing fish produced more rises. This effect rests on losing males emitting on average more rises than losing females in mixed-sex competitions (A) and males having higher EODfs than females. (C) In trials won by males (blue), the smaller the size difference to the winner (Δsize=size_loser_−size_winner_), the more rises were produced by the losing opponent of either sex. In trials won by females (red), the opposite effect was observed. This effect can, however, also result from overall higher rise rates and larger size differences of males compared with females when losing against females (A). Correlation coefficients and their significance are displayed in corresponding colors. (D) With increasing experience in the experiments, losing fish produced fewer rises per trial. *P≤0.05; n.s. not significant.

In trials won by males, the losing competitor of either sex produced less rises than in trials won by females (*U*=84.0, *P*=0.02; Fig. 4A). Consequently, the number of rises produced by losing fish correlated positively with the difference in EODfs, because of the sexually dimorphic EODf in *A. leptorhynchus* (*r*=0.32, *P*=0.049; Fig. 4B). Interestingly, in trials won by males the sex of the losing fish did not have an effect (*U*=37.0, *P*=0.36), whereas in trials won by females losing males produced more rises than losing females (*U*=10.0, *P*=0.036).

### Sex-specific dependence of rise emission on body size

The dependence on sex of the winner was even stronger when considering the contestants’ body size. In pairings won by males, the number of rises emitted by losers tended to increase with loser size (*r*=0.42, *P*=0.064) and decrease with winner size (*r*=−0.51, *P*=0.022). When regarding the difference in body size, the number of rises emitted by losers increased with its size approaching and exceeding the size of the winning male (*r*=0.74, *P*<0.001; Fig. 4C). Backward elimination in a multiple linear regression model of number of rises in dependence on absolute sizes of competitors and their difference resulted in size difference as the only parameter (*t*=4.72, *P*<0.001) remaining in the significant model (*F*_1,18_=22.3, *P*<0.001) with *R*^2^=0.55.

In pairings won by females, the number of rises emitted by losers decreased with loser size (*r*=−0.55, *P*=0.023) and was unaffected by winner size. When regarding the difference in body size, the number of detected loser rises decreased the more similar the competitors were in size (*r*=−0.75, *P*<0.001; Fig. 4C), i.e. the effect was opposite to the one found for trials won by males. In a multiple linear regression model for trials won by females, size difference was the only parameter (*t*=−4.36, *P*=0.001) remaining in the significant model (*F*_1,15_=18.98, *P*<0.001) with *R*^2^=0.56 after backward elimination.

### Habituation of rise rates and loser effects

The number of rises produced by losers was independent from the outcome of preceding competitions, rejecting a loser effect on the communication behavior of *A. leptorhynchus*. However, the fish's total experience in the experiment influenced the number of emitted rises. With increasing experience, i.e. the more trials a fish participated in, the quantity of detected loser rises decreased (*r*=−0.39, *P*=0.018) independently of the paired sexes (Fig. 4D). The time scale of this habituation matches the one reported for habituation of chirp emission in response to 60 s long stimulations with an EOD mimic (Harvey-Girard et al., 2010).

### Agonistic interactions

We detected in total 2480 chasing events and 804 agonistic interactions involving physical contact in the 19 trials where we recorded and evaluated these behaviors with IR video. Agonistic interactions were exclusively detected during the dark phase of each trial and stopped with or shortly after the onset of the light phase (Fig. 1E). In random visual inspections of videorecordings we found that agonistic behaviors were always initiated by those fish later identified as winners. Per trial, we observed on average 128±72 chase behaviors lasting 7.4±6.5 s and 36±21 physical contacts. The number of physical contacts tended to increase with the number of chasing events (*r*=0.37, *P*=0.12). Interestingly, none of the factors discussed so far had an impact on the number of agonistic interactions, including the competitor's sex, size and EODf differences, and their experience in the experiment. In particular, and similarly to the rises, the number of interactions per trial were highly variable (contacts: CV=0.55, chase events: CV=0.58) and neither the number of contacts (*r*=−0.27, *P*=0.24) nor the number of chase events (*r*=0.30, *P*=0.20) correlated with the number of rises.

### Sex-specific dependence of the duration of chase behaviors on body size differences

**Fig. 5.**
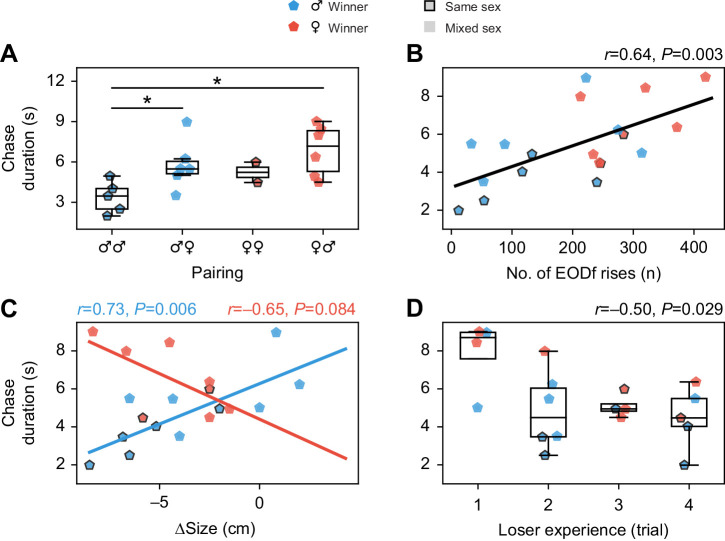
**Agonistic interactions.** While the number of agonistic interactions was independent of the physical characteristics of the opponents, the duration of chasing events showed similar dependencies as the number of rises. (A) The median chase duration was the shortest in male–male interactions. In the annotations of the sex pairings, the first symbol indicates the winner. (B) The median chase duration increased with the number of rises emitted by losers. (C) In trials won by males the median chase duration increased with losers approaching and exceeding the size of winners (Δsize=size_loser_−size_winner_). In trials won by females, the opposite effect was observed; however, similar explanations as for the quantity of rises in the respective pairings apply (Fig. 4C). Correlation coefficients and their significance are shown in corresponding colors. (D) With increasing experience in the experiments, the median chase duration decreased. *P≤0.05.

The duration of the chase behaviors was sensitive to differences in body size. The median duration of chasing event was shorter in male–male competitions compared with other pairings (*U*=5, *P*=0.003). For other pairings, the median durations of chasing events were indistinguishable (Fig. 5A).

In trials won by males, the median chase duration tended to decrease with winner size (*r*=−0.58, *P*=0.06) and was unaffected by size of the losing fish. However, it increased with the size of the losing fish relative to the size of the winner (*r*=0.73, *P*=0.006; Fig. 5C). Size difference remained after backward elimination the only parameter (*t*=3.54, *P*=0.006) in a linear regression model predicting median chasing duration (*F*_1,19_=12.5, *P*=0.006) with *R*^2^=0.58. In trials won by females, median chase duration was independent of absolute sizes but tended to decrease with decreasing size difference between competitors (*r*=−0.65, *P*=0.08; Fig. 5C). Size difference was the last size parameter excluded by backward elimination, but was not significant (*t*=−2.07, *P*=0.084, *R*^2^=0.42). Chase durations were not correlated with differences in EODf between the competitors (*r*=0.18, *P*=0.45).

### Habituation of the duration of chase behaviors

Beyond physical differences, the experience of the competitors in the experiment influenced the duration of chase behaviors. The duration of chase events decreased with the number of trials the losing fish participated in (*r*=−0.50, *P*=0.029; Fig. 5D). The number of chase behaviors was unaffected by experience (*r*=0.05, *P*=0.83). Finally, the observed communication behavior had an impact on the chasing duration. In trials where losers emitted more rises, chasing events lasted longer (*r*=0.64, *P*=0.003; Fig. 5B).

### Some rises triggered agonistic interactions

**Fig. 6.**
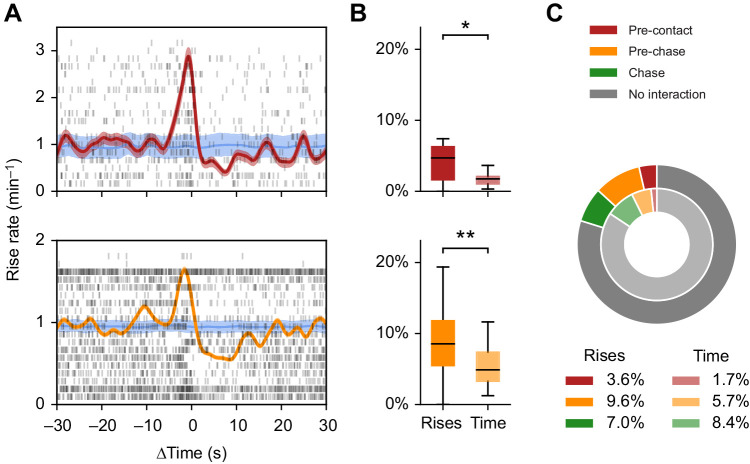
**Rises trigger other fish to initiate agonistic attacks.** (A) Rise times and rates relative to physical contacts (top) and the onset of chasing events (bottom). Each line of the rasters (black) shows rise times aligned to all agonistic events of a single competition trial. Corresponding rise rates (red/orange) were obtained by convolving rise times with Gaussian kernels with s.d. of 1 s and normalizing by the number of agonistic events and trials. 98% confidence intervals (pink/pale orange areas) were estimated by jack-knifing. The null hypothesis (mean in blue, 1st and 99th percentile in pale blue) was obtained by randomly permuting rise intervals. (B) Fraction of all rises within the dark phase of a trial that occurred within 5 s prior to agonistic contacts (top) or the onset of chase behaviors (bottom) and the corresponding times these 5 s windows make up relative to the total dark-phase lasting 3 h. (C) Mean fractions of rises (outer circle) occurring within 5 s prior to physical contacts (red), prior to the onset of chase behaviors (orange) and during chase events (green) and the corresponding times (inner circle) over all trials. *P≤0.05; **P≤0.01.

Within ∼5 s prior to agonistic interactions, rise rates accumulated over all agonistic interactions were increased (Fig. 6A,B). Because the baseline rate of rises was just one rise per minute, this does not imply that a burst of rises triggered agonistic interactions. Rather, the probability of a single rise to evoke an agonistic interaction was increased. In particular, chances for agonistic contacts were higher 0.7 s after a rise and chances for chase behaviors were higher 1.6 s after a rise. Consequently, the fraction of rises occurring within 5 s before agonistic onset exceeded the fraction expected from the corresponding times, the number of agonistic interactions times 5 s (agonistic contacts: 3.6% vs 1.7%, *t*=2.9, *P*=0.010; chase behaviors: 9.6% vs 5.7%, *t*=−3.1, *P*=0.007; Fig. 6B).

### Reduced rise rates during chase events

Rise rates were approximately halved during chase events (Fig. 6A, bottom). After the onset of chasing, the rise rate was reduced for ∼10 s, clearly outlasting the average duration of chase behaviors (7.4 s). Again, this just implies that the chances of observing a single rise during chasing is reduced. Approximately 7.0% of rises occurred during chase events, less than expected from the corresponding time covered by the chase events (8.4%, *t*=3.4, *P*=0.003; Fig. 6C).

### Most rises did not trigger agonistic interactions

All the rises emitted within 5 s prior to agonistic contacts and chase events, as well as during the chase events, make up 20% of all rises (Fig. 6C). Although this is disproportionately more than expected from the corresponding times, the majority of the rises (80%) could not be linked to any obvious interaction. Also, note that the fish engaged in actual agonistic interactions in the form of chase behaviors just 8.4% of the time. Vice versa, agonistic interactions were not exclusively triggered by rises emitted by losers. Rises preceded 15.6% of all chase events and 19.4% of physical contacts, respectively.

## DISCUSSION

In staged competition experiments between pairs of the electric fish *A. leptorhynchus* of either sex, we recorded electrocommunication signals, so-called ‘rises’, and agonistic behaviors. Losers were characterized by relatively smaller body sizes and their continuously higher rise emission rates. Rises were costly for losers since they raised the chance of being attacked and being chased for longer by winners. Our results suggest that rises signal an individual's motivation to continue opponent assessment by stimulating ritualized fighting behaviors.

### Body size as a proxy for RHP in *A. leptorhynchus*

An animal's ability to win fights, its RHP, is often correlated to body size or weight, because physical strength is usually directly related to size (Parker, 1974; Archer, 1988). Difference in body size was also the best predictor for the outcome of competitions in our experiments (Fig. 2, Fig. S3). Thus, competitors seem to be capable of assessing each other's body size, even in darkness. Electric field amplitudes have been shown to correlate with body size across electric fish species (*Eigenmannia virescens*: Westby and Kirschbaum, 1981; *Sternopygus*: Hopkins, 1972; *A. albifrons*: Knudsen, 1975). The lateral line organ might provide further sensory cues to assess a contestant's body size (Butler and Maruska, 2015).

The outcome of competitions can also be influenced by sex-dependent differences in motivation (Enquist and Leimar, 1987; Arnott and Elwood, 2008; Dunham, 2008). In males, an increased motivation and likelihood to compete has often been observed (Archer, 1988). In our experiments, males won competitions slightly more often than females, but mainly because of a bias of males being larger than females in these trials (Fig. 2). Overall, average body size was independent of sex (Fig. S1A). However, when evaluated separately, the correlation between body size and winning was more pronounced in trials won by males than trials won by females (Fig. 2B–E). This could reflect a higher valuation of suitable shelters by males.

### Relevant signals and interactions of *A. leptorhynchus* during competition

Competitors assess their own and/or their opponent's RHP based on cues, including actively emitted signals, and adapt their behavior accordingly (Clutton-Brock et al., 1979; Enquist et al., 1990; Payne, 1998; Arnott and Elwood, 2009). Properties of the continuously emitted electric field of *A. leptorhynchus*, in particular EODf, could be utilized in opponent assessment. Males increase both EODf and the androgen 11-ketotestosterone at the transition to the breeding season (Cuddy et al., 2012) and males with higher EODf seem to fertilize more eggs (Hagedorn and Heiligenberg, 1985; Henninger et al., 2018).

Outside the breeding season, males with higher EODf have been found to be more territorial during the day (Raab et al., 2019) and to occupy their preferred shelter alone (Dunlap and Oliveri, 2002). Whereas in the latter study EODf was weakly but significantly correlated with body size, we did not have such a correlation (Fig. S1A). Consequently, in our experiments, the predictive power of EODf on competition outcome was insignificant (Fig. S2), demonstrating only a minor role for EODf signaling RHP in addition to body size.

The RHP of contestants usually affects their fighting behavior, e.g. quantity, intensity, duration or point of giving up (Arnott and Elwood, 2009; Taylor et al., 2001; Briffa and Elwood, 2004). While in *A. leptorhynchus* the number of agonistic interactions did not correlate with any of the measured parameters, the duration of chase events was dependent on the difference in contestant's body size, the main factor determining RHP, and the winner's sex (discussed below). Interestingly, the number of rises emitted by losers was correlated in similar ways to the contestant's RHP. This similarity, together with the observation of rises frequently triggering agonistic attacks, suggests that rises play a role in assessing opponents.

### Electrocommunication with rises

*A. leptorhynchus* has been shown to use a rich repertoire of electrocommunication signals in social interactions (Smith, 2013; Benda, 2020). Some of the various types of chirps, transient elevations of EODf within less than ∼500 ms, are used in agonistic same-sex encounters to deter agonistic attacks (Hupé and Lewis, 2008; Henninger et al., 2018) and in courtship, where synchronization of spawning is probably only one of their many functions (Hagedorn and Heiligenberg, 1985; Triefenbach and Zakon, 2003; Cuddy et al., 2012; Henninger et al., 2018). In contrast, evidence for the function of rises is scarce and inconsistent. Rises are characterized by smaller but much longer increases in EODf in comparison to chirps (Hopkins, 1974; Hagedorn and Heiligenberg, 1985). They vary considerably in their size (a few up to several tens of Hertz) and over three orders of magnitude in their duration (less than a second to up to a few minutes, Tallarovic and Zakon, 2002). The large number of rises we detected in our experiments clearly formed a continuous distribution of sizes and durations, and we found no indication of distinct functional roles of rises of different sizes (Triefenbach and Zakon, 2008), refuting earlier attempts to categorize rises (Hagedorn and Heiligenberg, 1985; Tallarovic and Zakon, 2002; Dye, 1987).

### Function of rises

Rises have been observed to be followed by attacks or bouts of aggression, both in *Eigenmannia* (Hopkins, 1974) and *A. leptorhynchus* (Triefenbach and Zakon, 2008), and to primarily be emitted by subordinates in *Apteronotus albifrons* (Serrano-Fernández, 2003). We also found rises in *A. leptorhynchus* to be primarily emitted by losing fish (Fig. 3A,B) and agonistic interactions to be more frequent after the emission of rises (Fig. 6A). These common findings further support the hypothesis of rises being conserved signals in gymnotiform electric fish (Turner et al., 2007).

As only ∼20% of rises were followed by agonistic interactions, one could argue that rises are submissive signals aiming to avert upcoming agonistic attacks. However, the emission of more rises did not decrease the number of agonistic interactions and even increased the duration of chasing events (Fig. 5B), suggesting that rises rather encourage agonistic interactions than deter them. This contradicts the interpretation of rises as submissive signals (Hopkins, 1974; Serrano-Fernández, 2003) and as a general expression of stress (Smith, 2013). Chirps, in contrast, have been shown to reduce attack probability in competition experiments (Hupé et al., 2008). *A. leptorhynchus* thus use a variety of electrocommunication signals of different meanings in social interactions.

Communication signals in general aim to alter the behavior of a receiver in a net beneficial fashion for the sender and they are only produced when the potential benefits outweigh the costs (Endler, 1993; Seyfarth et al., 2010). In contests, they can convey information about physical condition and RHP (Davies and Halliday, 1978; Clutton-Brock et al., 1979), social status (Huyghe et al., 2005; Fernald, 2014), and motivation or behavioral intent (e.g. aggression: Triefenbach and Zakon, 2008; Kareklas et al., 2019; or submission: Hupé and Lewis, 2008; Batista et al., 2012) and often already convey sufficient information to settle competitions without the necessity of escalating costly fights (Arnott and Elwood, 2009). In our experiments, winners could reliably be predicted within the initial 25 min of each trial based on the number of rises emitted by either fish (Fig. 3C). Nevertheless, losers continued to emit more rises than the winner until the end of the dark phase (Fig. 3D). We never observed a switch in communication behavior between contestants (Fig. 3E). Therefore, rises were apparently not used to ultimately win competitions. What then is the purpose of rises?

### Sexual dimorphic behavioral traits

Male *A. leptorhynchus* have been shown to be more territorial than females (Dunlap and Oliveri, 2002) and show more intense dominance displays (Raab et al., 2019). Females, in contrast, are more tolerant to the presence of conspecifics (Zupanc and Maler, 1993; Cuddy et al., 2012). All these observations could be explained by an increased resource valuation in males (territoriality at shelters) in comparison to females, and males being more motivated to win competitions. Our data further support this hypothesis, as discussed below.

In trials won by males, both the number of rises and the duration of chase events increased with decreasing size difference between contestants (Figs 4C, 5C). This is not unusual, because of increased chances of success when competing with opponents of similar size (e.g. Clutton-Brock et al., 1979; Enquist et al., 1990). A higher motivation of males could be inferred by losers by means of behavioral cues and interpreted as potentially higher costs when engaging in competition, which could reduce a loser's motivation to compete. This could explain the overall lower rise production by losers in trials won by males (Fig. 4A) and the resulting shorter chase events (Fig. 5A).

In trials won by females, we found the opposite relationship. With decreasing size difference fewer rises were produced by the losing fish and chase duration decreased (Figs 4C, 5C). These negative correlations, however, are mainly carried by the sex of the losing fish. Males losing against females tended to be much smaller (Fig. 2E) and at the same time emit more rises (Fig. 4A) and interact longer during chase events (Fig. 5A) compared with all other pairings. Females competing against females were more similar in size (Fig. 2C, Fig. S1A), emitted fewer rises (Fig. 4A) and had shorter chase events (Fig. 5A). The higher intrinsic motivation of males in addition to the lower potential costs in competing with less territorial females could explain the enhanced rise production in males regardless of RHP of female opponents.

In other species, the mere presence of a potential mating partner often affects communication (e.g. Barske et al., 2015) and other behaviors associated with reproductive success (Taylor, 1975). Accordingly, the specific sex pairing could evoke males to emit disproportionately more rises when losing against females. Males could additionally be motivated to continue assessment in order to indicate increased fighting capabilities and appear more suitable as potential mating partner. However, winning males not emitting more rises towards females and females responding with equal levels of aggression to rises of both sexes (Fig. 5) rather argues against rises to signal a male's quality to females in our competition experiments.

Nevertheless, competition between *A. leptorhynchus* and associated behaviors presumably change with reproductive state. The motivation of males to compete could be enhanced, especially for same-sex rivals. Females could use male rises to assess their capability and motivation to compete and thus their quality. Testing these speculations, however, requires extensive breeding experiments.

In summary, the dependence of rise production on the fish's RHP and the link between agonistic interactions and rise emission support our hypothesis of rises signaling an individual's motivation to continue opponent assessment using ritualized fighting. The fact that both behaviors are also dependent on the competitor's sex additionally suggests sexually dimorphic behavioral traits in *A. leptorhynchus*, potentially arising from a higher motivation of males to win competitions despite substantial differences in RHP.

### Mutual assessment in *A. leptorhynchus*

Analysing the dependence of competition behaviors on the contestants’ RHP allows to differentiate between assessment strategies, i.e. pure self-, cumulative or mutual assessment (Arnott and Elwood, 2009). In cumulative assessment, costs arising from an individual's own actions during competitions and those being inflicted by opponents are accumulated until an endurance threshold is reached and the animal retreats (Payne, 1998). In our experiments, however, this threshold never seems to be reached, because rise emission and agonisitic interactions went on throughout the dark phase. For pure self-assessment, a positive correlation between both contestants’ absolute RHP and the extent of behaviors associated with competition is expected (Taylor et al., 2001). This can be rejected, because absolute body size of neither winner nor loser predicted competition outcome (Fig. 2F), and both the number of rises and duration of chasing events rather decreased with winner size.

Both, communication and agonistic behaviors remained steady throughout single trials. Both are presumably low-cost behaviors, because no injuries or other negative consequences resulted from them. This supports mutual assessment where low-cost competition behaviors are repetitively performed in order to accurately assess an opponent's RHP relative to their own (e.g. Clutton-Brock et al., 1979). Furthermore, animals are expected to improve in accuracy of assessing the opponent with increasing experience (Enquist et al., 1990; Grosenick et al., 2007). This matches previous observations on decreasing competition intensity over trials in another gymnotiform electric fish (Westby and Box, 1970) as well as our own observations on *A. leptorhynchus* where the number of emitted rises and the duration of chasing events decrease with the fish's experience in the competition experiment (Figs 4D, 5D).

### Dominance in *A. leptorhynchus*

Competitions are not exclusively used to directly secure access to resources, but also to establish dominance hierarchies, that indirectly regulate access to resources (Wauters and Dhondt, 1992; Sapolsky, 2005; Taves et al., 2009). After establishing dominance, knowledge about an individual's social status can prevent costly repetitive fighting and therefore can be beneficial for all individuals involved (Fernald, 2014; Huyghe et al., 2005). Characteristics of social hierarchies and behavioral correlates of dominance vary widely across species (Clutton-Brock et al., 1979; Cigliano, 1993; Sapolsky, 2005). In group living species, beyond regulating access to resources, social hierarchies often occur with complex social dynamics, such as leader–follower dynamics (e.g. Strandburg-Peshkin et al., 2018; Janson, 1990). In solitary species, in contrast, dominance is primarily associated with resource-based benefits (Cigliano, 1993).

Dominance hierarchies have also been suggested for *A. leptorhynchus* (Hagedorn and Heiligenberg, 1985; Dunlap and Oliveri, 2002). Since behavioral observations suggest that *A. leptorhynchus* is a solitary living species (Stamper et al., 2010; Raab et al., 2019; Henninger et al., 2020), this dominance can be assumed to be mainly resource based.

Previous studies suggest that male but not female *A. leptorhynchus* form a dominance hierarchy (Hagedorn and Heiligenberg, 1985; Dunlap and Oliveri, 2002). Indeed, as discussed above, males seemed to be more motivated to win competitions. Nevertheless, competition outcome was independent of the contestant's sex and mainly determined by relative body size (Fig. S3). Dominance in *A. leptorhynchus* thus appears to be sex-independent, in line with similar studies on other gymnotiform electric fish (Batista et al., 2012; Zubizarreta et al., 2020).

### Rises in the social hierarchy of *A. leptorhynchus*

In social hierarchies, dominants often use agonistic attacks to keep subordinates under control (Clutton-Brock et al., 1979; Creel et al., 1996; Janson, 1985). In *A. leptorhynchus*, subordinates could emit rises to signal their motivation to continue assessment, with the aim to reduce relative dominance (e.g. Kareklas et al., 2019). Dominants, in contrast, could counteract with agonistic attacks. The interplay and balance between rises and agonistic attacks could define the relative dominance between contestants and regulate skewness in access to resources. This would imply that motivation of the fish in the competition depends on the valuation of not only present but also regularly encountered resources, most likely food, that were absent during our experiments.

This hypothesis on a possible benefit of continuous rise emission by losers is supported by a single exceptional trial, where the dominant fish shared the superior shelter with the subordinate at the end of a trial. In this mixed-sex trial, the smaller male (11.9 cm) was continuously emitting 180 rises during the dark phase and apparently succeeded in reducing the relative dominance difference to the larger female (12.5 cm, no rises during dark phase) by gaining access to the shelter.

### Conclusion

Male *A. leptorhynchus* seem to be more motivated to win staged competitions for a superior shelter than females. Nevertheless, contest outcomes were mainly determined by relative body size, reflecting the contestants’ overall fighting ability, their RHP. During competition, *A. leptorhynchus* interact physically by means of ritualized fights and use rises as distinct electrocommunication signals. The extent of both behaviors depends on the contestants’ RHP, suggesting that *A. leptorhynchus* assess their opponents during contests (mutual assessment). Here, rises are almost exclusively emitted by losers and seem to signal their motivation to continue physical assessment. Rises triggered agonistic attacks and enhanced the duration of chase events. The motivation to continue assessment could reflect a loser's attempt to reduce relative dominance, which is counteracted by dominant fish with agonistic attacks.

## Supplementary Material

Supplementary information
