# Supplementary material for: Electrocommunication signals indicate motivation to compete during dyadic interactions of an electric fish
Source: J Exp Biol. 2021 Oct 6;224(19):jeb242905. doi: 10.1242/jeb.242905 (PMC8541736; doi:10.1242/jeb.242905)
Supplement: Supplementary information [file jexbio-224-242905-s1.pdf]

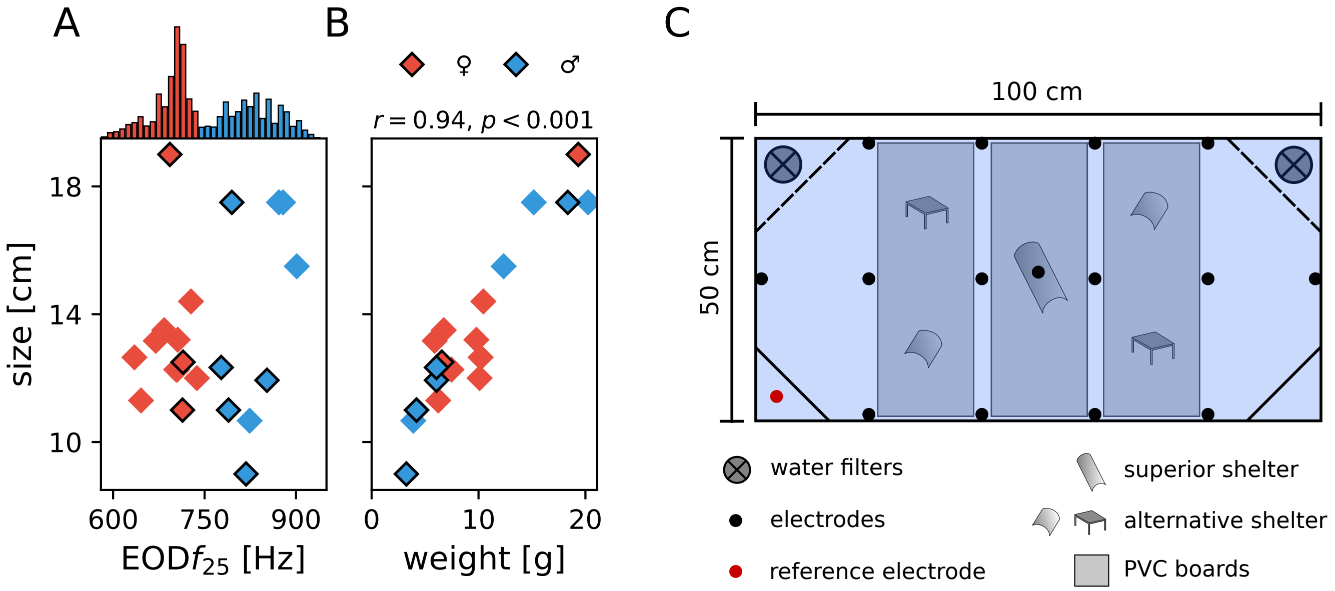

Fig. S1. Physical characteristics of the fish and experimental setup. **A** EODf and size (length) of each of the 21 *A. leptorhynchus* participating in the experiments. EODf was not correlated with size, neither for all fish nor within the sexes. EODfs were temperature corrected to 25 °C. Males (blue) were identified by their higher EODfs compared to female EODfs (red). The histogram on top shows the distribution of EODfs of either sex measured in all trials for every five minutes. Black marker edges indicate fish whose sex has been verified by gonadal inspection. **B** Weight and size of fish were independent of sex and increase proportional to each other. **C** The competition tank was equipped with one high quality shelter (center tube) and four low quality shelters (two short tubes and two tables) attached to PVC-boards. A total of 15 electrodes (black circles) were distributed in the tank to record electric behaviors of interacting fish. Two air-powered water-filters were placed in the corners behind PVC boards with netted windows (dashed lines). The other two corners were shielded with PVC boards (black lines), one of them contained the reference electrode (red circle).

Journal of Experimental Biology • Supplementary information

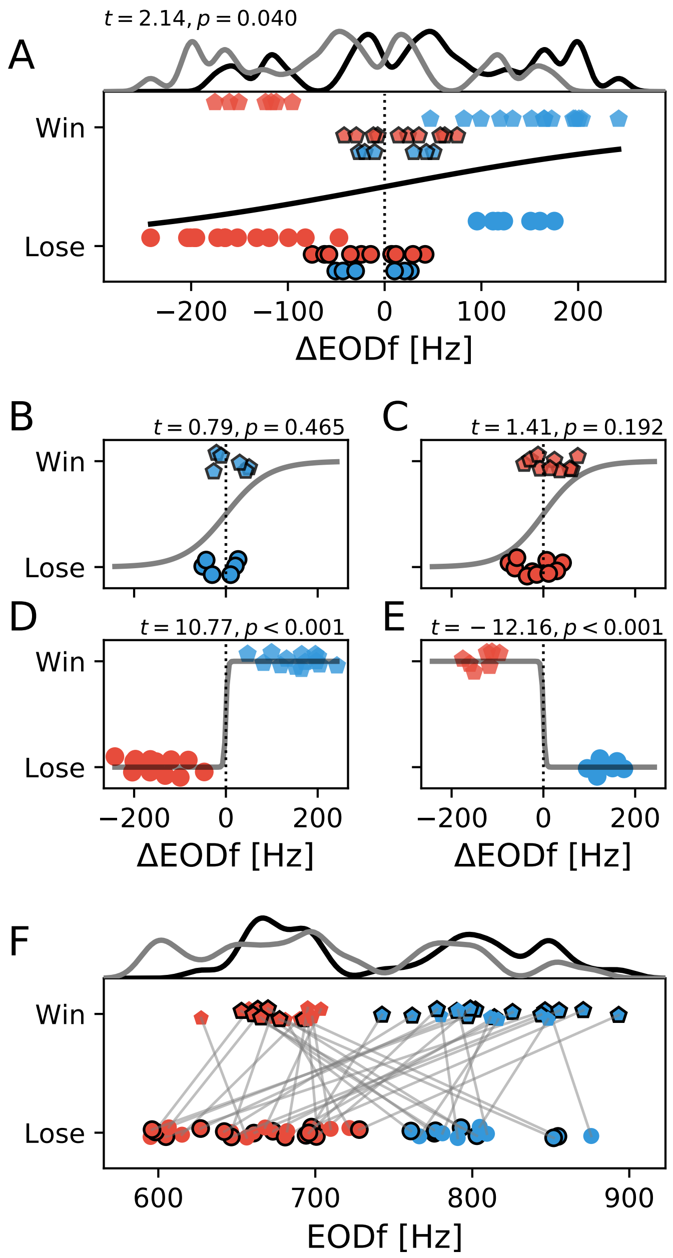

Fig. S2. EOD frequencies of winners and losers. Same annotation scheme as in Fig. 2. **A** Winners tend to have higher EODfs than their opponents. **B, C** Winners of same-sex encounters do not have significantly h igher E ODfs t han losers (AUC=73 %). **D, E** In mixed-sex competitions the sexual dimorphic EODf does not predict competition outcome. More males ($n = 14$) were winning mixed-sex trials than females ($n = 7$), explaining the overall trend of winners having higher EODfs than losers (panel A). **F** Absolute EODf of winners and losers convey even less information about the outcome of the competitions (AUC=65 %).

Journal of Experimental Biology • Supplementary information

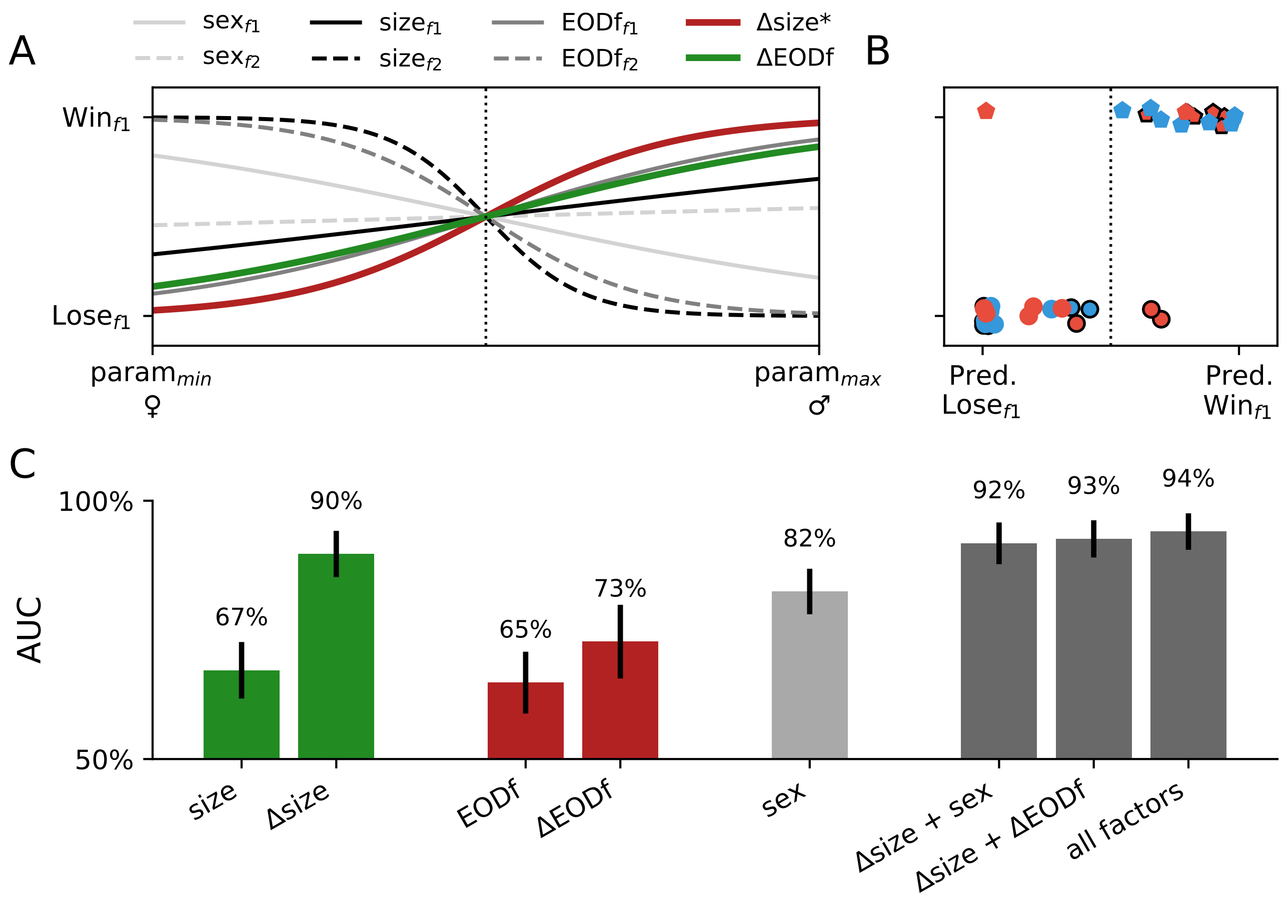

Fig. S3. Logistic regression models predicting the outcome of competitions. **A** Visual representation of the impact of all factors (sex, absolute size, and EODf of fish $f1$ and $f2$, as well as their differences in size and EODf) on the generalized linear model with a logistic link function, corresponding to table 1. The steeper the logistic functions the more discriminative the respective factor. Size difference, Δsize, is the only significant factor. **B** Predictions of the GLM on fish_1 of each of the 37 competition trials being the winner or loser based on all factors shown in A. Same marker code as in Fig. 2. **C** Area under the curve (AUC, with standard deviation estimated by bootstrapping) extracted from a receiver-operating-characteristic (ROC) analysis to quantify the performance of single factors and combinations of factors to discriminate winners of competitions from losers. Chance level is at $\text{AUC} = 50\,\%$.

Journal of Experimental Biology • Supplementary information
